# The changing trend and prediction of the burden of anxiety disorder in Chinese population from 2022 to 2035

**DOI:** 10.3389/fpubh.2025.1580771

**Published:** 2025-04-03

**Authors:** Ruiheng Fan, Ciming Pan, Hui Fang, Jida Zhang

**Affiliations:** ^1^Zhejiang Chinese Medical University, Hangzhou, Zhejiang, China; ^2^Yunnan Provincial Hospital of Traditional Chinese Medicine, Kunming, Yunnan, China; ^3^Anhui University of Chinese Medicine, Hefei, Anhui, China

**Keywords:** anxiety, disease burden, epidemiology, China, GBD

## Abstract

**Objective:**

To analyze the disease burden of anxiety disorders globally, in middle SDI countries, and in China using GBD public database.

**Methods:**

Data on anxiety disorders were extracted from the GBD database, and GBDR_V2.36 was used for data visualization and plotting.

**Results:**

From 1990 to 2021, global anxiety disorder cases and prevalence rose steadily. In 2021, the 30–34 age group had the highest global cases (male:female≈3:7), while DALY rates peaked in 25–29-year-olds. Medium-SDI countries followed similar trends, with peak DALYs in the 20–24 age group. China saw declining prevalence/incidence but rising DALYs, with highest cases in 50–54-year-olds (male:female≈3:5) and DALY rates in 75–84-year-olds. Globally, medium-SDI countries, and China, incidence peaked in 10–14-year-olds. Projections suggest China’s anxiety disorder burden will surge through 2035.

**Conclusion:**

Globally and in middle SDI countries, prevalence, incidence, and DALY rates increased, with the 30–34 age group showing the highest case burden (women outnumbering men) and peak DALY rates in younger adults (25–29 globally, 20–24 in middle SDI). In contrast, China saw a decline in prevalence and incidence, with the burden peaking later (50–54 for cases, 75–84 for DALYs). Projections indicate rising anxiety disorder burden in China by 2035, necessitating region- and age-specific interventions.

## 1 Introduction

Anxiety disorders encompass generalized anxiety disorder, panic disorder, and social anxiety disorder. Both psychotherapy and pharmacological treatment serve as effective therapeutic approaches, demonstrating efficacy rates ranging from 60 to 85% (1). The pathogenesis of anxiety is associated with physiological and psychological factors, as well as familial, religious, social, and behavioral influences (2). Trigger factors such as academic stress, environmental changes, and societal transitions may exacerbate symptoms, significantly impacting patients and their families (3). Since the COVID-19 outbreak in 2019, global cases of anxiety disorders have surged dramatically, with long-term sequelae like tinnitus showing heightened correlation with anxiety disorders (4). Anxiety disorders have substantially compromised quality of life and become a source of significant distress (5). Given their escalating impact, our study aims to systematically elucidate the global burden of anxiety disorders, with particular focus on medium-SDI countries and China. By leveraging the Global Burden of Disease (GBD) dataset, this research presents a comprehensive overview of anxiety disorders’ prevalence, incidence, mortality, and disability-adjusted life years (DALYs). Furthermore, we conduct rigorous age-stratified analyses to facilitate the development of precise prevention and treatment strategies by governmental and healthcare institutions.

## 2 Data extraction and analysis workflow

### 2.1 Data sources and scope

Database: Disease burden data for anxiety disorders were extracted from the Global Burden of Disease (GBD) public database (Version 2023), covering global populations, middle SDI countries, and China.

Time span: Historical trend analysis (1990–2021); Predictive modeling (2022–2035).

Population definitions: Global populations, middle SDI countries, and China, stratified by sex (male/female) and age-standardized groups.

### 2.2 Data extraction and variable definitions

Core indicators: Disease burden metrics included prevalence, incidence, and disability-adjusted life years (DALYs).

Incidence: New cases per population during a specific period.

Prevalence: Total cases (new and existing) per population at a specific time.

DALYs: Sum of years of life lost (YLLs) and years lived with disability (YLDs).

### 2.3 Extraction tools

Data were filtered using the GBD Results Tool^1^ to extract population-specific datasets for global, middle SDI countries, and China. Raw datasets were exported in CSV format.

### 2.4 Statistical analysis methods

Analyses were performed using the GBDR_V2.36 software^2^ to: Quantify the contributions of age, period, and cohort effects to changes in anxiety disorder burden. Integrate historical trends and demographic shifts to project anxiety disorder burden from 2022 to 2035. Conduct visualization analysis. All statistical analyses and data visualizations were performed using R (version 4.3.3) and JD_GBDR (V2.24, Jingding Medical Technology Co., Ltd.).

BAPC Models for Projections: (Bayesian Age-Period-Cohort) models provide a comprehensive framework for making projections using integrated nested Laplace approximations (INLA) for full Bayesian inference. Key features of BAPC models include: (1) Generation of age-specific and age-standardized projected rates. (2) Automatic addition of Poisson noise when interest lies in the predictive distribution. BAPC is particularly useful for projecting future rates based on historical data, making it an invaluable tool for public health planning and analysis (Analysis Tools Help Page.^3^ For detailed parameters and explanations, refer to the article: PMID: 28139001).

## 3 Results

### 3.1 Prevalence trends (1990–2021)

**Figure 1:**
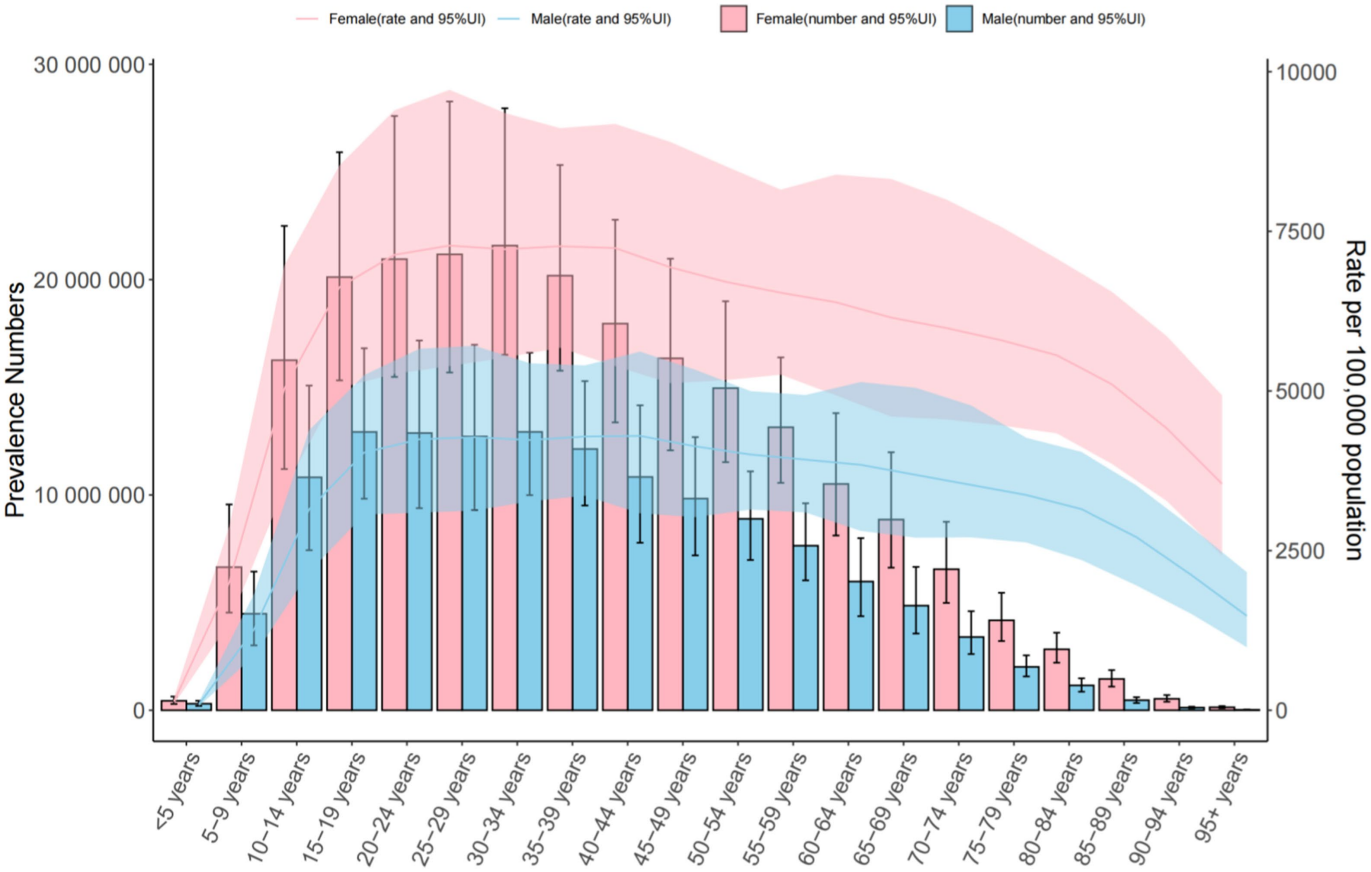
Trends in the number and prevalence of anxiety disorders in the global from 1990 to 2021.

**Figure 2:**
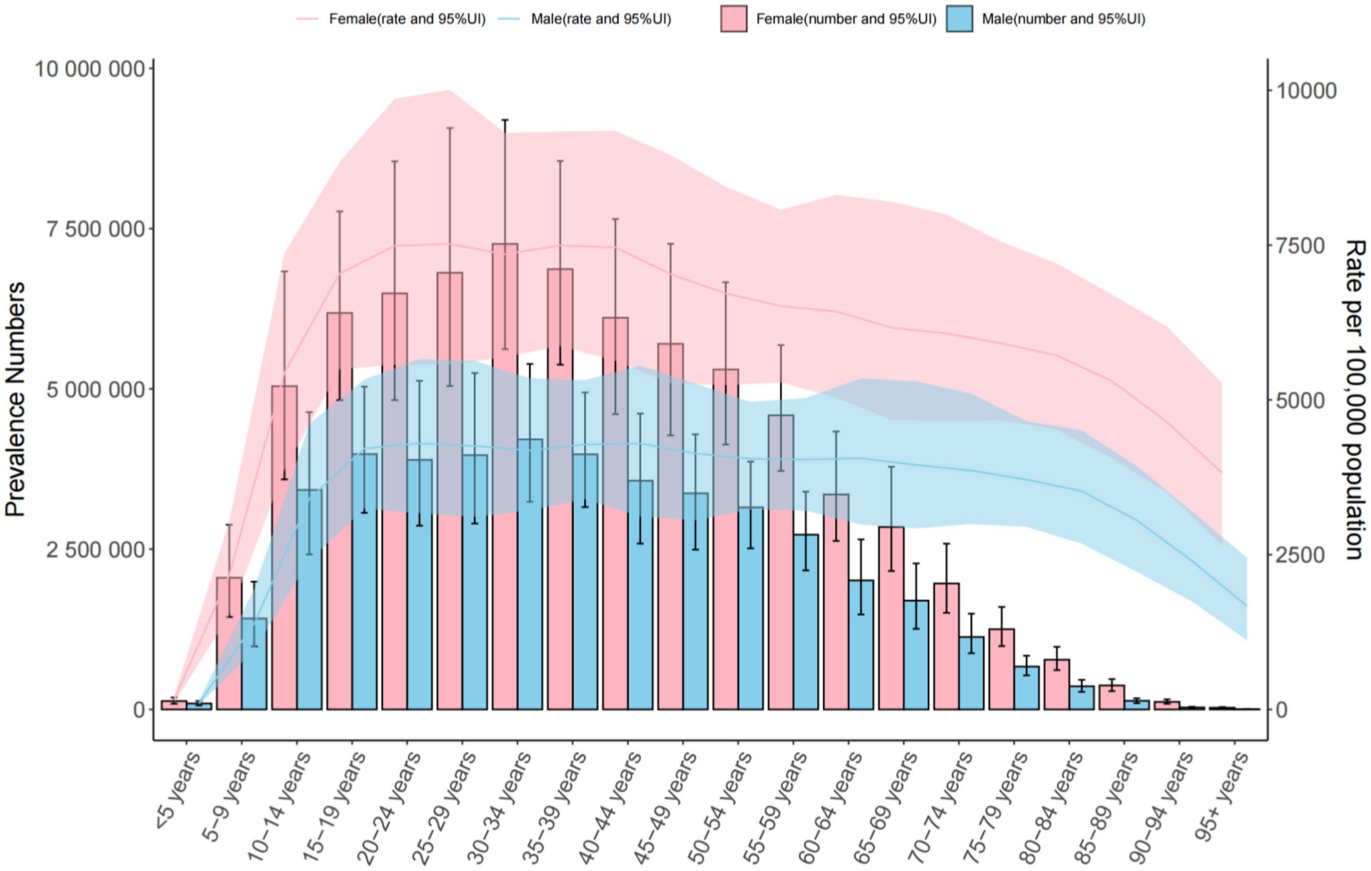
Trends in the number and prevalence of anxiety disorders in middle SDI countries from 1990 to 2021.

**Figure 3:**
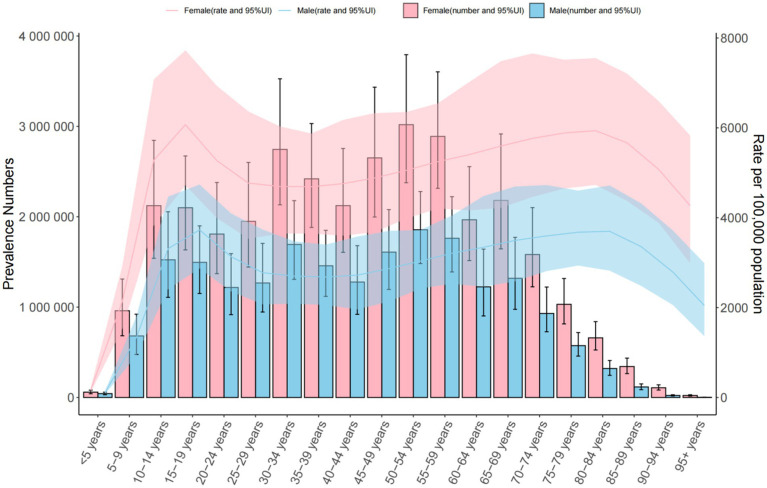
Trends in the number and prevalence of anxiety disorders in China from 1990 to 2021.

Global: Prevalence increased from 3,617.356 (1990) to 4,551.986 per 100,000 (2021). The 30–34 age group had the highest cases (34,509,154.849 [95% UI = 26,618,309.601–44,568,217.725]), with a male-to-female ratio of 3:7. Middle SDI Countries: Prevalence rose from 3,442.221 to 3,734.496 per 100,000. The 30–34 age group predominated (male-to-female ratio: 4:7). China: Prevalence declined (3,572.654 to 4,782.196 per 100,000), with the highest burden in the 50–54 age group (male-to-female ratio: 3:5) (Table 1 and Figures 1–3).

**Table 1:** Prevalence trends of anxiety disorders in different age groups in the global, SDI countries and China from 1990 to 2021.

District	Age group (years)	Male						Female						Both					
		1990		2021		AAPC		1990		2021		AAPC		1990		2021		AAPC	
		Prevalence rate (1/100,000)	95%UI	Prevalence rate (1/100,000)	95%UI	AAPC	95%CI	Prevalence rate (1/100,000)	95%UI	Prevalence rate (1/100,000)	95%UI	AAPC	95%CI	Prevalence rate (1/100,000)	95%UI	Prevalence rate (1/100,000)	95%UI	AAPC	95%CI
Global	<5	68.655	(46.309, 98.325)	89.349	(59.078, 129.661)	0.356	(0.154, 0.557)	104.246	(71.214, 150.780)	137.701	(91.835, 200.420)	0.389	(0.183, 0.595)	85.901	(58.377, 123.853)	112.725	(74.842, 164.092)	0.375	(0.171, 0.579)
	5–14	1784.875	(1246.473, 2485.379)	2191.338	(1497.905, 3077.963)	0.165	(−0.008, 0.337)	2820.66	(1990.737, 3868.286)	3494.183	(2408.910, 4874.166)	0.161	(−0.016, 0.339)	2289.489	(1605.310, 3159.527)	2822.22	(1943.616, 3947.092)	0.16	(−0.015, 0.335)
	15–19	3394.057	(2631.384, 4396.739)	4035.269	(3066.994, 5248.536)	0.118	(−0.046, 0.282)	5516.486	(4268.109, 7031.599)	6625.558	(5045.434, 8535.001)	0.123	(−0.042, 0.288)	4438.221	(3419.448, 5702.685)	5295.792	(4031.416, 6858.024)	0.11	(−0.054, 0.274)
	20–24	3445.022	(2561.447, 4568.033)	4243.156	(3093.259, 5660.231)	0.203	(0.017, 0.390)	5771.82	(4285.209, 7623.164)	7132.57	(5270.134, 9395.553)	0.184	(0.004, 0.364)	4599.395	(3421.341, 6052.171)	5664.507	(4179.255, 7486.072)	0.183	(0.004, 0.363)
	25–29	3460.505	(2563.385, 4587.557)	4276.685	(3128.013, 5708.483)	0.26	(0.071, 0.450)	5883.609	(4375.879, 7825.462)	7276.708	(5390.693, 9716.473)	0.203	(0.019, 0.387)	4665.431	(3481.103, 6197.656)	5760.459	(4262.687, 7650.862)	0.225	(0.044, 0.406)
	30–34	3505.575	(2686.222, 4516.650)	4231.295	(3269.800, 5435.017)	0.26	(0.081, 0.438)	5972.194	(4592.861, 7687.149)	7219.215	(5523.087, 9353.105)	0.18	(0.022, 0.339)	4722.231	(3646.773, 6054.581)	5708.898	(4403.504, 7372.983)	0.213	(0.055, 0.373)
	35–39	3522.123	(2756.565, 4464.420)	4286.629	(3359.713, 5400.339)	0.279	(0.111, 0.448)	5944.755	(4667.415, 7409.062)	7265.996	(5676.923, 9115.700)	0.198	(0.043, 0.353)	4715.083	(3713.180, 5892.669)	5762.34	(4493.485, 7218.511)	0.236	(0.085, 0.387)
	40–44	3606.799	(2588.753, 4740.239)	4296.738	(3084.752, 5616.611)	0.225	(0.077, 0.373)	6049.937	(4557.559, 7702.565)	7238.076	(5389.118, 9182.152)	0.145	(−0.028, 0.318)	4802.653	(3579.444, 6170.673)	5755.438	(4259.945, 7300.566)	0.187	(0.032, 0.341)
	45–49	3608.072	(2647.039, 4704.107)	4132.942	(3024.085, 5333.170)	0.146	(0.032, 0.261)	5991.791	(4465.616, 7820.454)	6936.622	(5122.279, 8899.444)	0.074	(−0.085, 0.234)	4776.35	(3506.602, 6197.114)	5528.211	(4034.761, 7096.098)	0.116	(−0.016, 0.249)
	50–69	3498.886	(2747.155, 4371.568)	3887.45	(3083.302, 4916.576)	0.086	(−0.012, 0.185)	5779.754	(4655.825, 7315.247)	6480.651	(5273.440, 8199.812)	0.004	(−0.146, 0.154)	4654.621	(3728.920, 5869.111)	5209.676	(4233.508, 6533.896)	0.041	(−0.080, 0.163)
	70–74	3304.115	(2503.004, 4462.224)	3528.53	(2708.704, 4773.105)	0.079	(−0.017, 0.175)	5573.504	(4224.796, 7462.796)	5985.389	(4555.676, 7998.142)	−0.012	(−0.157, 0.132)	4565.119	(3500.752, 6093.564)	4834.879	(3694.877, 6463.735)	0.022	(−0.096, 0.139)
	75–84	3071.289	(2400.821, 3916.695)	3287.265	(2560.563, 4150.965)	0.078	(0.028, 0.128)	5451.24	(4203.942, 6985.196)	5696.838	(4426.932, 7343.878)	−0.125	(−0.226, −0.025)	4505.553	(3506.235, 5718.363)	4638.021	(3611.864, 5917.910)	−0.141	(−0.223, −0.059)
	85–89	2541.137	(1831.878, 3329.566)	2710.371	(1954.430, 3520.748)	0.159	(0.114, 0.203)	4914.504	(3720.072, 6312.288)	5106.742	(3860.486, 6556.082)	−0.067	(−0.141, 0.006)	4119.135	(3089.782, 5282.214)	4202.495	(3174.058, 5350.602)	−0.101	(−0.170, −0.031)
	90–94	2019.32	(1427.528, 2763.006)	2114.48	(1506.598, 2863.786)	0.162	(0.117, 0.208)	4289.113	(3158.352, 5700.630)	4413.137	(3275.821, 5857.056)	−0.053	(−0.114, 0.007)	3622.262	(2663.742, 4820.118)	3664.216	(2706.578, 4901.511)	−0.075	(−0.128, −0.022)
	95+	1458.962	(958.387, 2150.395)	1477.912	(986.665, 2162.242)	0.123	(0.076, 0.170)	3556.568	(2413.758, 4950.621)	3545.878	(2424.668, 4932.558)	−0.115	(−0.168, −0.062)	3020.403	(2063.284, 4200.418)	2972.176	(2040.381, 4130.079)	−0.139	(−0.184, −0.095)
Middle SDI	<5	78.572	(54.598, 111.070)	100.911	(68.497, 142.338)	0.217	(0.008, 0.427)	114.777	(79.606, 162.348)	154.522	(105.125, 219.264)	0.396	(0.201, 0.592)	96.017	(66.744, 136.314)	126.587	(86.274, 179.392)	0.319	(0.118, 0.520)
	5–14	1980.938	(1413.876, 2691.650)	2385.746	(1675.601, 3244.316)	0.046	(−0.154, 0.247)	3016.572	(2156.964, 4083.288)	3789.698	(2702.330, 5159.877)	0.195	(−0.001, 0.391)	2485.364	(1777.932, 3373.815)	3059.412	(2177.793, 4179.524)	0.123	(−0.073, 0.321)
	15–19	3559.705	(2785.158, 4527.204)	4215.826	(3245.546, 5329.179)	0.024	(−0.149, 0.198)	5588.649	(4360.619, 7087.937)	7041.167	(5493.551, 8844.842)	0.206	(0.031, 0.382)	4558.303	(3553.129, 5753.447)	5576.941	(4322.012, 7027.309)	0.119	(−0.053, 0.292)
	20–24	3422.513	(2569.504, 4476.468)	4298.715	(3164.836, 5660.018)	0.175	(−0.026, 0.375)	5611.925	(4181.105, 7344.437)	7489.286	(5568.241, 9865.284)	0.329	(0.127, 0.531)	4507.143	(3391.575, 5895.110)	5858.835	(4363.394, 7695.553)	0.267	(0.073, 0.460)
	25–29	3337.159	(2485.769, 4396.234)	4255.726	(3111.398, 5630.252)	0.311	(0.105, 0.517)	5602.071	(4182.000, 7502.846)	7519.451	(5568.074, 10010.715)	0.411	(0.204, 0.619)	4460.955	(3341.625, 5909.757)	5864.443	(4322.637, 7822.778)	0.381	(0.188, 0.575)
	30–34	3364.242	(2589.997, 4314.694)	4181.87	(3215.766, 5350.096)	0.368	(0.157, 0.580)	5679.855	(4411.719, 7280.249)	7353.291	(5692.225, 9311.645)	0.44	(0.279, 0.601)	4498.589	(3488.400, 5738.016)	5751.831	(4435.351, 7304.368)	0.423	(0.260, 0.586)
	35–39	3377.166	(2647.609, 4266.907)	4280.842	(3395.940, 5318.336)	0.41	(0.198, 0.621)	5613.829	(4401.085, 6987.082)	7494.666	(5868.887, 9336.235)	0.488	(0.334, 0.642)	4469.943	(3524.312, 5593.997)	5876.174	(4596.220, 7266.673)	0.472	(0.316, 0.629)
	40–44	3486.436	(2524.885, 4558.226)	4296.131	(3114.261, 5555.939)	0.329	(0.160, 0.498)	5749.369	(4325.291, 7365.230)	7465.049	(5629.284, 9347.710)	0.384	(0.191, 0.577)	4578.254	(3423.109, 5868.460)	5868.902	(4429.745, 7406.797)	0.386	(0.226, 0.547)
	45–49	3538.251	(2628.395, 4564.738)	4135.22	(3057.686, 5262.475)	0.218	(0.096, 0.341)	5736.785	(4295.872, 7375.984)	7030.829	(5271.468, 8955.311)	0.247	(0.058, 0.437)	4596.897	(3415.435, 5917.938)	5579.165	(4168.475, 7097.540)	0.267	(0.127, 0.406)
	50–69	3644.284	(2921.083, 4537.667)	4029.185	(3218.788, 5019.509)	0.029	(−0.081, 0.139)	5652.9	(4586.614, 7097.309)	6497.391	(5336.131, 8019.730)	0.036	(−0.146, 0.217)	4632.739	(3744.771, 5799.622)	5287.871	(4315.872, 6561.829)	0.06	(−0.074, 0.194)
	70–74	3628.611	(2800.959, 4805.240)	3859.666	(2995.997, 5101.680)	0.017	(−0.090, 0.125)	5576.291	(4286.500, 7439.440)	6073.155	(4658.627, 7995.623)	0.013	(−0.167, 0.193)	4673.536	(3628.479, 6154.012)	5021.436	(3884.614, 6598.164)	0.037	(−0.096, 0.171)
	75–84	3402.127	(2682.267, 4246.113)	3645.088	(2876.023, 4552.375)	0.024	(−0.031, 0.078)	5430.647	(4259.099, 6884.043)	5836.588	(4631.056, 7408.920)	−0.165	(−0.318, −0.012)	4559.141	(3587.325, 5732.517)	4853.31	(3867.617, 6111.978)	−0.132	(−0.247, −0.016)
	85–89	2724.652	(1992.281, 3546.979)	3056.089	(2223.756, 3933.851)	0.293	(0.240, 0.346)	4885.64	(3733.981, 6201.958)	5303.204	(4056.934, 6702.930)	−0.006	(−0.134, 0.122)	4041.717	(3085.943, 5125.229)	4438.497	(3351.058, 5578.993)	0.076	(−0.038, 0.190)
	90–94	2101.079	(1502.616, 2841.239)	2400.66	(1746.892, 3198.222)	0.445	(0.404, 0.485)	4198.986	(3098.415, 5594.608)	4642.679	(3506.180, 6182.436)	0.141	(0.044, 0.238)	3412.25	(2521.284, 4572.499)	3881.088	(2926.047, 5160.720)	0.292	(0.207, 0.377)
	95+	1479.065	(957.265, 2180.011)	1666.654	(1114.438, 2448.261)	0.421	(0.405, 0.437)	3352.184	(2268.573, 4666.145)	3818.225	(2645.698, 5267.252)	0.331	(0.277, 0.385)	2721.916	(1846.355, 3799.711)	3128.45	(2185.214, 4320.509)	0.398	(0.358, 0.437)
China	<5	88.368	(61.746, 121.987)	103.607	(72.914, 140.752)	0.216	(−0.046, 0.480)	137.785	(96.881, 191.001)	164.038	(115.986, 225.836)	0.496	(0.299, 0.694)	111.591	(78.295, 154.643)	131.646	(92.842, 180.582)	0.377	(0.159, 0.595)
	5–14	2231.817	(1621.421, 3003.633)	2274.698	(1641.781, 3066.572)	0.022	(−0.350, 0.396)	3522.141	(2565.136, 4691.790)	3625.228	(2645.117, 4855.899)	0.169	(−0.191, 0.530)	2853.533	(2070.204, 3846.612)	2905.774	(2095.447, 3916.880)	0.077	(−0.283, 0.438)
	15–19	3645.227	(2848.940, 4637.737)	3730.939	(2871.257, 4738.321)	−0.208	(−0.354, −0.062)	5770.68	(4546.098, 7331.066)	6070.999	(4721.731, 7726.930)	−0.141	(−0.315, 0.033)	4679.447	(3701.958, 5900.460)	4814.892	(3709.325, 6083.077)	−0.212	(−0.379, −0.044)
	20–24	3132.21	(2393.608, 4038.461)	3132.71	(2358.903, 4095.859)	−0.578	(−0.754, −0.402)	5145.185	(3880.838, 6616.695)	5271.336	(3994.286, 6934.724)	−0.707	(−0.899, −0.516)	4115.617	(3139.911, 5313.434)	4135.374	(3142.106, 5438.170)	−0.673	(−0.840, −0.506)
	25–29	2786.681	(2082.857, 3716.346)	2777.865	(2073.120, 3738.301)	−0.681	(−0.989, −0.372)	4722.732	(3527.760, 6253.475)	4769.68	(3531.802, 6364.318)	−0.978	(−1.228, −0.727)	3728.6	(2786.452, 4943.206)	3719.08	(2766.823, 4978.888)	−0.855	(−1.094, −0.615)
	30–34	2659.348	(2040.036, 3453.803)	2701.703	(2085.159, 3475.167)	−0.524	(−0.842, −0.204)	4565.88	(3523.070, 5825.674)	4695.195	(3646.325, 6031.438)	−0.885	(−1.131, −0.637)	3571.508	(2753.642, 4575.551)	3663.777	(2817.174, 4720.933)	−0.731	(−0.971, −0.491)
	35–39	2677.645	(2102.177, 3407.129)	2681.166	(2058.177, 3402.471)	−0.331	(−0.598, −0.063)	4582.445	(3597.715, 5714.177)	4689.517	(3646.412, 5874.698)	−0.666	(−0.864, −0.468)	3597.584	(2819.489, 4541.333)	3659.101	(2857.979, 4587.724)	−0.524	(−0.702, −0.346)
	40–44	2740.095	(1976.765, 3574.293)	2722.1	(1960.781, 3580.771)	−0.152	(−0.384, 0.081)	4676.783	(3522.025, 6016.029)	4759.19	(3600.878, 6176.952)	−0.451	(−0.611, −0.291)	3661.305	(2715.078, 4745.285)	3714.863	(2757.672, 4872.421)	−0.316	(−0.439, −0.194)
	45–49	2861.726	(2153.053, 3653.026)	2869.103	(2132.361, 3708.685)	−0.145	(−0.366, 0.078)	4777.735	(3569.369, 6207.203)	4886.391	(3680.462, 6328.396)	−0.398	(−0.567, −0.229)	3766.675	(2812.933, 4833.255)	3861.277	(2891.280, 4995.171)	−0.27	(−0.403, −0.138)
	50–69	3211.177	(2540.334, 4012.779)	3236.264	(2553.108, 4082.059)	−0.387	(−0.606, −0.168)	5183.47	(4202.466, 6489.173)	5288.894	(4243.850, 6665.097)	−0.609	(−0.847, −0.371)	4162.084	(3373.955, 5211.375)	4261.851	(3420.367, 5295.426)	−0.491	(−0.681, −0.300)
	70–74	3541.045	(2762.639, 4638.186)	3595.802	(2812.865, 4726.831)	−0.379	(−0.588, −0.170)	5650.246	(4341.257, 7495.984)	5765.698	(4462.778, 7656.449)	−0.623	(−0.866, −0.380)	4672.257	(3628.080, 6106.016)	4712.984	(3663.367, 6219.249)	−0.505	(−0.698, −0.312)
	75–84	3592.24	(2862.494, 4474.698)	3685.205	(2918.034, 4616.356)	−0.145	(−0.211, −0.080)	5753.545	(4586.982, 7161.870)	5905.775	(4723.067, 7528.270)	−0.54	(−0.784, −0.297)	4854.583	(3882.920, 6029.492)	4886.896	(3912.966, 6184.406)	−0.476	(−0.654, −0.298)
	85–89	3279.389	(2442.046, 4210.423)	3355.818	(2481.641, 4318.651)	0.049	(−0.063, 0.162)	5495.187	(4257.550, 6932.331)	5667.404	(4374.456, 7207.622)	−0.335	(−0.566, −0.104)	4774.54	(3686.655, 5980.736)	4822.701	(3710.671, 6095.057)	−0.296	(−0.492, −0.100)
	90–94	2701.892	(1985.684, 3613.304)	2792.032	(2059.022, 3718.064)	0.149	(−0.011, 0.310)	4883.927	(3675.111, 6356.239)	5077.708	(3888.947, 6597.334)	−0.222	(−0.448, 0.006)	4304.47	(3272.037, 5628.858)	4447.168	(3390.303, 5742.173)	−0.18	(−0.411, 0.051)
	95+	2074.296	(1376.977, 2976.266)	2045.629	(1362.801, 2988.193)	0.233	(0.043, 0.423)	4234.591	(3009.329, 5650.276)	4266.255	(2987.148, 5829.769)	−0.17	(−0.344, 0.004)	3820.093	(2726.491, 5114.102)	3846.021	(2703.996, 5249.079)	−0.122	(−0.317, 0.073)

### 3.2 Incidence trends (1990–2021)

**Figure 4:**
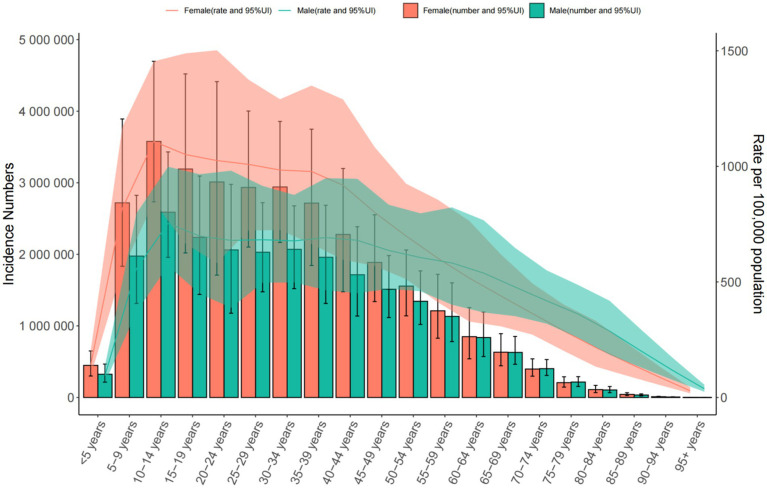
Trends in the incidence and prevalence of anxiety disorders in the global from 1990 to 2021.

**Figure 5:**
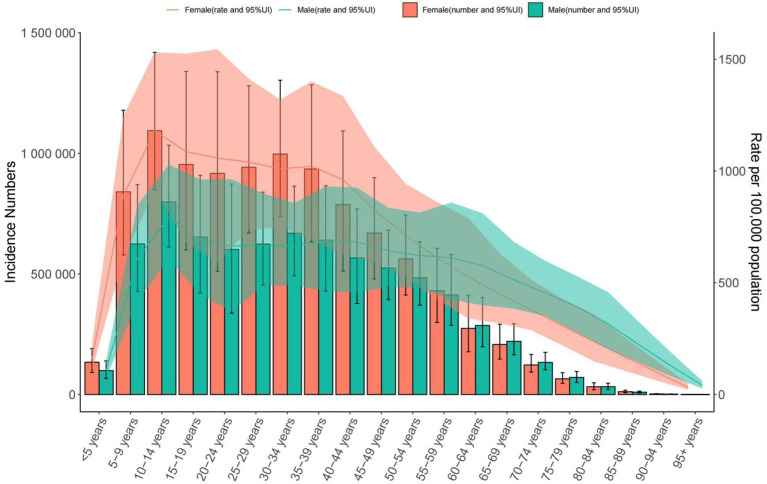
Trends in the incidence and prevalence of anxiety disorders in middle SDI countries from 1990 to 2021.

**Figure 6:**
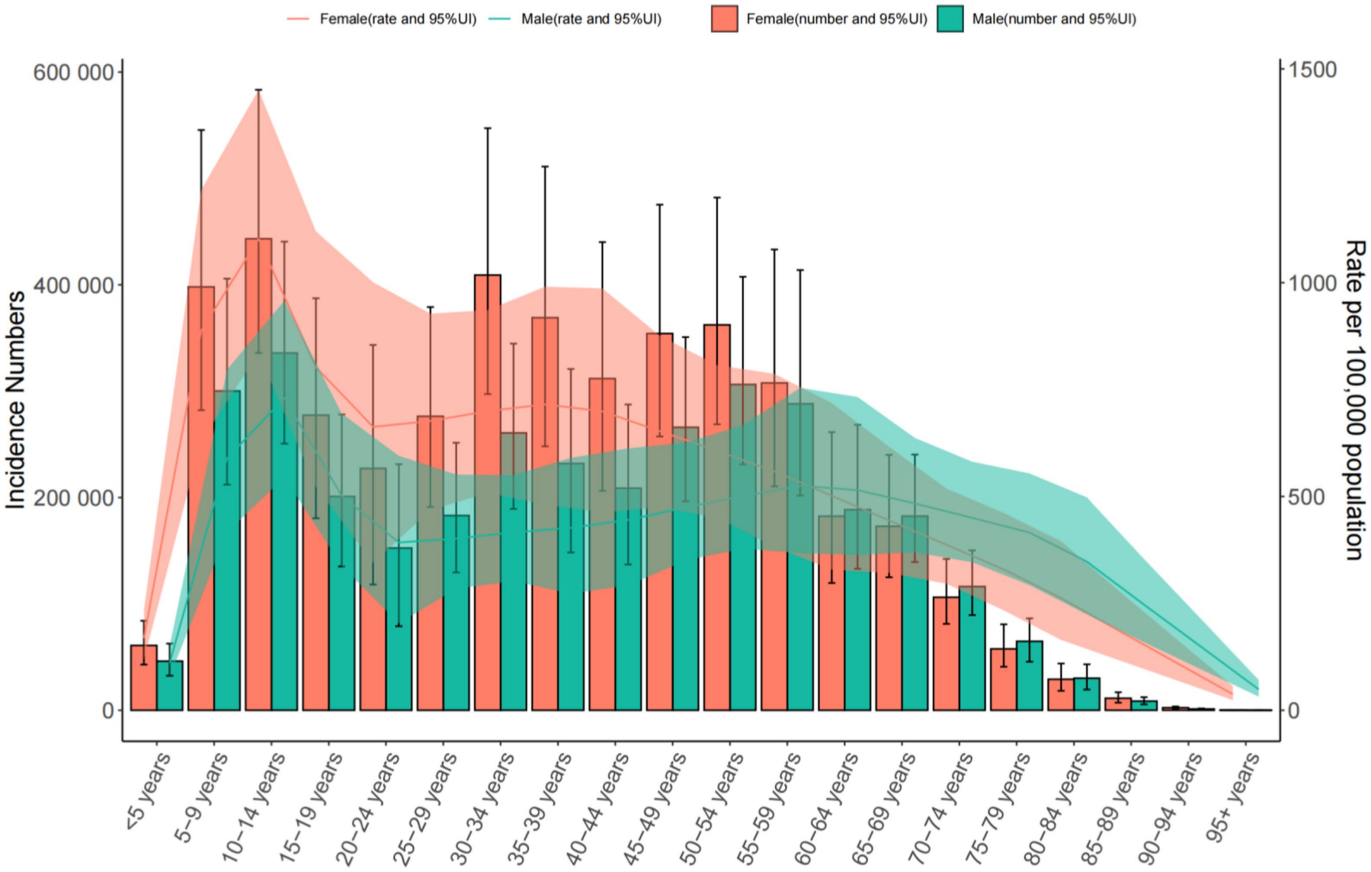
Trends in the incidence and prevalence of anxiety disorders in China from 1990 to 2021.

Global: Incidence increased from 566.957 to 683.246 per 100,000. The 10–14 age group had the highest rates (925.073 [95% UI = 704.079–1,213.413] per 100,000). Middle SDI Countries: Incidence rose from 578.919 to 712.126 per 100,000. China: Incidence declined slightly (542.095 to 543.570 per 100,000), peaking in the 10–14 age group (904.013 [95% UI = 681.091–1,184.287] per 100,000) (Table 2 and Figures 4–6).

**Table 2:** Trends in the incidence of anxiety disorders in different age groups in the global, SDI countries and China from 1990 to 2021.

District	Age group (years)	Male						Female						Both					
		1990		2021		AAPC		1990		2021		AAPC		1990		2021		AAPC	
		Incidence rate (1/100,000)	95%UI	Incidence rate (1/100,000)	95%UI	AAPC	95%CI	Incidence rate (1/100,000)	95%UI	Incidence rate (1/100,000)	95%UI	AAPC	95%CI	Incidence rate (1/100,000)	95%UI	Incidence rate (1/100,000)	95%UI	AAPC	95%CI
Global	<5	73.42	(49.586, 104.051)	95.537	(63.062, 137.404)	0.388	(0.183, 0.594)	106.948	(72.631, 153.771)	141.1	(94.315, 204.412)	0.388	(0.183, 0.594)	89.667	(60.626, 128.621)	(94.315, 204.412)	117.565	0.374	(0.171, 0.578)
	5–14	653.777	(467.492, 897.972)	530.258	(381.795, 718.701)	0.196	(0.026, 0.366)	960.737	(698.107, 1311.650)	775.256	(565.887, 1039.733)	0.194	(0.023, 0.366)	802.418	(578.026, 1101.231)	(565.887, 1039.733)	649.616	0.193	(0.022, 0.364)
	15–19	698.614	(449.534, 965.980)	555.413	(361.983, 764.858)	0.249	(0.050, 0.449)	1051.349	(665.041, 1488.873)	843.151	(536.902, 1181.211)	0.213	(0.023, 0.404)	870.267	(553.655, 1221.542)	(536.902, 1181.211)	696.97	0.219	(0.025, 0.413)
	20–24	679.707	(388.065, 981.435)	522.358	(289.779, 765.134)	0.334	(0.115, 0.555)	1025.168	(582.004, 1502.341)	803.961	(448.446, 1186.566)	0.251	(0.043, 0.459)	849.645	(485.170, 1243.389)	(448.446, 1186.566)	662.067	0.278	(0.068, 0.489)
	25–29	682.576	(496.561, 915.307)	540.984	(388.863, 728.488)	0.344	(0.153, 0.534)	1008.715	(722.229, 1375.475)	812.34	(574.557, 1112.328)	0.248	(0.066, 0.431)	843.88	(612.895, 1142.702)	(574.557, 1112.328)	675.92	0.287	(0.105, 0.470)
	30–34	677.529	(497.736, 876.385)	557.12	(409.215, 726.735)	0.319	(0.155, 0.484)	984.101	(724.546, 1290.474)	815.486	(596.784, 1082.358)	0.241	(0.094, 0.388)	829.136	(612.640, 1081.758)	(596.784, 1082.358)	684.558	0.276	(0.128, 0.424)
	35–39	692.106	(464.186, 948.731)	564.72	(375.015, 773.857)	0.283	(0.151, 0.415)	977.71	(664.023, 1349.542)	806.72	(540.897, 1114.093)	0.283	(0.151, 0.415)	833.569	(561.263, 1141.107)	(540.897, 1114.093)	683.887	0.313	(0.183, 0.442)
	40–44	680.197	(451.325, 946.226)	570.93	(375.683, 781.335)	0.253	(0.116, 0.390)	918.18	(595.918, 1289.676)	771.987	(495.690, 1075.950)	0.242	(0.103, 0.381)	798.22	(519.946, 1107.710)	(495.690, 1075.950)	669.342	0.254	(0.124, 0.384)
	45–49	635.978	(469.571, 833.702)	558.25	(409.530, 731.419)	0.129	(0.013, 0.245)	800.887	(568.832, 1083.239)	695.203	(491.344, 945.776)	0.146	(0.019, 0.273)	718.046	(524.792, 959.419)	(491.344, 945.776)	625.372	0.145	(0.030, 0.260)
	50–69	560.279	(413.145, 761.985)	510.083	(376.543, 696.684)	0.041	(−0.061, 0.144)	580.304	(416.559, 807.674)	524.12	(375.761, 723.904)	0.042	(−0.075, 0.159)	570.489	(420.767, 785.190)	(375.761, 723.904)	517.195	0.042	(−0.063, 0.147)
	70–74	418.257	(319.397, 550.158)	394.032	(302.113, 510.852)	0.04	(−0.020, 0.100)	363.186	(271.804, 493.363)	338.283	(250.066, 456.740)	−0.012	(−0.096, 0.072)	388.975	(294.985, 512.893)	(250.066, 456.740)	363.055	0.029	(−0.041, 0.098)
	75–80	361.012	(258.325, 486.747)	338.183	(241.917, 456.536)	0.093	(0.055, 0.132)	287.653	(201.166, 401.122)	267.776	(187.975, 374.688)	0.035	(−0.022, 0.092)	320.908	(229.466, 441.427)	(187.975, 374.688)	296.637	0.096	(0.050, 0.143)
	81–84	286.948	(185.217, 418.210)	269.411	(174.182, 388.614)	0.13	(0.098, 0.162)	216.677	(133.530, 332.075)	201.756	(125.551, 307.364)	0.073	(0.033, 0.114)	246.084	(155.515, 365.212)	(125.551, 307.364)	227.16	0.147	(0.116, 0.178)
	85–89	200.694	(129.607, 293.122)	191.076	(123.259, 275.770)	0.107	(0.072, 0.143)	152.722	(93.998, 234.218)	143.023	(88.777, 217.438)	0.063	(0.022, 0.104)	170.824	(107.987, 253.915)	(88.777, 217.438)	159.127	0.125	(0.095, 0.155)
	90–94	116.888	(75.528, 171.037)	113.555	(73.147, 164.244)	0.08	(0.044, 0.116)	89.63	(55.244, 137.373)	85.03	(52.679, 129.564)	0.04	(0.003, 0.076)	98.511	(62.754, 146.965)	(52.679, 129.564)	93.411	0.083	(0.054, 0.112)
	95+	37.726	(24.492, 55.261)	37.708	(24.337, 54.624)	0.035	(−0.004, 0.073)	29.052	(17.947, 44.458)	28.311	(17.534, 43.408)	−0.015	(−0.047, 0.016)	31.458	(20.148, 47.023)	(17.534, 43.408)	30.713	0.026	(0.004, 0.048)
Middle SDI	<5	84.059	(58.019, 117.313)	107.932	(73.276, 151.574)	0.399	(0.205, 0.593)	117.888	(81.341, 167.337)	158.548	(108.376, 225.187)	0.399	(0.205, 0.593)	100.359	(69.113, 141.390)	132.174	(90.094, 187.071)	0.319	(0.119, 0.520)
	5–14	701.027	(514.151, 938.722)	578.986	(422.141, 783.661)	0.088	(−0.085, 0.262)	1033.278	(761.629, 1372.162)	820.277	(606.155, 1084.747)	0.246	(0.085, 0.407)	860.453	(633.031, 1150.984)	696.512	(510.009, 935.194)	0.17	(0.004, 0.336)
	15–19	691.019	(446.367, 962.224)	540.934	(356.177, 743.307)	0.214	(0.005, 0.424)	1086.335	(682.674, 1525.419)	823.7	(522.922, 1146.138)	0.31	(0.112, 0.508)	881.464	(559.903, 1242.235)	680.105	(440.575, 940.161)	0.257	(0.057, 0.458)
	20–24	664.07	(372.351, 963.575)	484.79	(262.524, 716.688)	0.413	(0.172, 0.655)	1058.325	(589.380, 1544.569)	766.767	(413.918, 1133.682)	0.385	(0.150, 0.621)	856.852	(478.567, 1246.989)	624.481	(336.879, 918.671)	0.393	(0.161, 0.625)
	25–29	669.364	(487.678, 900.239)	508.394	(361.026, 686.912)	0.466	(0.262, 0.670)	1040.299	(738.825, 1413.083)	786.727	(557.749, 1075.400)	0.388	(0.184, 0.594)	852.201	(615.689, 1148.797)	646.497	(460.216, 882.574)	0.425	(0.228, 0.622)
	30–34	664.176	(488.361, 857.716)	530.617	(392.455, 692.593)	0.458	(0.269, 0.647)	1009.955	(747.283, 1319.927)	806.424	(591.888, 1066.503)	0.378	(0.224, 0.532)	835.349	(617.158, 1087.725)	665.726	(487.277, 875.084)	0.417	(0.261, 0.574)
	35–39	688.465	(461.885, 931.334)	539.918	(356.061, 737.416)	0.414	(0.274, 0.555)	1020.831	(691.341, 1401.298)	807.085	(541.125, 1102.614)	0.414	(0.274, 0.555)	853.45	(574.066, 1164.443)	670.449	(442.575, 906.816)	0.454	(0.313, 0.596)
	40–44	682.385	(454.239, 926.044)	555.464	(361.380, 761.243)	0.343	(0.192, 0.495)	962.151	(625.891, 1335.729)	784.849	(510.626, 1083.917)	0.313	(0.157, 0.469)	821.236	(540.595, 1127.537)	666.138	(435.019, 912.355)	0.341	(0.199, 0.483)
	45–49	643.308	(482.835, 835.603)	556.01	(413.239, 723.004)	0.153	(0.029, 0.277)	825.775	(591.046, 1108.822)	709.656	(505.804, 953.561)	0.148	(0.009, 0.287)	734.298	(539.561, 967.817)	629.994	(463.547, 829.236)	0.165	(0.043, 0.287)
	50–69	590.142	(442.940, 797.693)	539.407	(402.600, 729.946)	−0.036	(−0.150, 0.078)	595.644	(428.500, 822.470)	546.97	(397.728, 756.658)	−0.062	(−0.190, 0.067)	592.948	(439.882, 808.869)	543.129	(403.249, 742.788)	−0.049	(−0.165, 0.067)
	70–74	454.75	(351.100, 597.914)	430.021	(330.549, 550.609)	−0.013	(−0.081, 0.055)	379.351	(288.353, 514.256)	363.115	(271.045, 483.945)	−0.128	(−0.217, −0.039)	415.176	(317.178, 549.362)	394.126	(300.816, 510.685)	−0.063	(−0.139, 0.013)
	75–80	393.921	(278.522, 530.059)	374.085	(267.464, 501.246)	0.073	(0.038, 0.109)	307.853	(219.761, 427.594)	296.307	(209.813, 407.703)	−0.07	(−0.126, −0.014)	347.427	(247.485, 474.340)	330.423	(236.460, 447.180)	0.02	(−0.023, 0.064)
	81–84	316.186	(203.875, 458.635)	296.608	(192.692, 431.191)	0.154	(0.125, 0.182)	237.109	(148.642, 359.628)	227.741	(142.867, 348.201)	−0.009	(−0.058, 0.041)	271.143	(170.658, 398.355)	256.131	(163.144, 372.414)	0.089	(0.053, 0.125)
	85–89	221.232	(142.856, 321.898)	205.707	(133.219, 298.109)	0.191	(0.164, 0.219)	168.118	(105.696, 254.064)	160.228	(101.032, 245.286)	0.039	(−0.009, 0.086)	188.557	(118.731, 277.320)	177.989	(113.496, 260.349)	0.106	(0.074, 0.138)
	90–94	128.8	(82.579, 189.442)	119.061	(76.877, 172.593)	0.241	(0.226, 0.257)	99.678	(62.511, 150.663)	94.438	(59.393, 144.915)	0.104	(0.069, 0.138)	109.57	(69.338, 161.480)	103.672	(66.732, 152.521)	0.127	(0.108, 0.146)
	95+	41.099	(26.518, 61.008)	38.694	(25.017, 56.760)	0.211	(0.198, 0.225)	32.744	(20.792, 49.655)	31.429	(19.952, 48.272)	0.109	(0.095, 0.124)	35.422	(22.898, 52.355)	33.874	(21.748, 50.259)	0.134	(0.124, 0.145)
China	<5	94.555	(66.207, 131.170)	110.848	(77.990, 150.570)	0.509	(0.313, 0.704)	142.143	(99.517, 197.911)	169.084	(119.109, 233.508)	0.509	(0.313, 0.704)	116.919	(82.116, 162.158)	137.869	(96.640, 189.993)	0.383	(0.166, 0.600)
	5–14	656.212	(478.501, 871.478)	635.286	(464.393, 853.414)	0.081	(−0.174, 0.337)	989.499	(727.355, 1317.523)	947.25	(697.686, 1267.545)	0.283	(0.059, 0.508)	811.951	(593.322, 1080.292)	785.6	(578.692, 1045.180)	0.169	(−0.061, 0.401)
	15–19	501.537	(337.347, 693.827)	485.789	(324.024, 660.938)	−0.417	(−0.574, −0.260)	802.156	(521.850, 1119.845)	767.136	(495.354, 1062.460)	−0.556	(−0.729, −0.383)	640.788	(421.598, 883.877)	622.689	(407.396, 856.631)	−0.54	(−0.698, −0.382)
	20–24	392.414	(203.295, 595.156)	383.908	(196.233, 585.683)	−0.751	(−1.201, −0.300)	662.767	(344.527, 1001.153)	645.908	(331.302, 981.981)	−1.186	(−1.530, −0.841)	519.165	(271.594, 783.641)	511.904	(262.835, 778.933)	−1.031	(−1.382, −0.680)
	25–29	401.414	(284.126, 551.351)	396.332	(275.896, 546.779)	−0.503	(−0.860, −0.145)	676.392	(467.797, 927.618)	666.243	(467.994, 918.033)	−0.84	(−1.079, −0.600)	531.353	(372.104, 730.851)	527.648	(371.155, 729.011)	−0.7	(−0.948, −0.452)
	30–34	416.021	(302.106, 549.922)	407.803	(301.170, 541.435)	−0.252	(−0.519, 0.016)	699.784	(508.439, 935.964)	682.004	(497.539, 905.351)	−0.52	(−0.693, −0.346)	552.967	(399.447, 736.530)	538.992	(388.530, 713.378)	−0.401	(−0.573, −0.228)
	35–39	426.739	(272.839, 590.211)	424.004	(273.675, 589.033)	−0.26	(−0.396, −0.123)	715.353	(481.008, 990.701)	700.284	(464.926, 965.460)	−0.26	(−0.396, −0.123)	567.275	(370.678, 787.567)	557.436	(363.981, 765.381)	−0.167	(−0.275, −0.059)
	40–44	445.038	(292.032, 612.596)	443.735	(289.042, 610.604)	−0.035	(−0.208, 0.139)	699.107	(462.580, 986.721)	689.093	(453.131, 958.591)	−0.178	(−0.305, −0.051)	568.857	(376.640, 786.709)	560.443	(368.346, 769.023)	−0.103	(−0.192, −0.015)
	45–49	474.549	(350.321, 625.827)	468.727	(350.533, 608.200)	−0.188	(−0.378, 0.001)	652.853	(474.279, 876.080)	644.957	(469.383, 856.834)	−0.26	(−0.393, −0.126)	562.245	(415.904, 748.490)	551.962	(411.987, 722.864)	−0.211	(−0.332, −0.089)
	50–69	507.11	(373.977, 693.932)	498.95	(371.100, 675.668)	−0.412	(−0.614, −0.209)	539.421	(389.897, 735.516)	538.835	(390.387, 748.654)	−0.414	(−0.583, −0.245)	523.254	(386.317, 702.534)	518.18	(387.608, 699.195)	−0.409	(−0.574, −0.243)
	70–74	449.979	(346.031, 581.345)	441.363	(338.391, 569.412)	−0.138	(−0.211, −0.064)	387.131	(295.928, 518.473)	385.514	(291.997, 503.808)	−0.268	(−0.380, −0.155)	417.621	(320.373, 547.253)	411.41	(317.494, 533.220)	−0.189	(−0.275, −0.104)
	75–80	415.502	(292.565, 553.911)	406.906	(289.276, 546.867)	0.037	(−0.016, 0.090)	329.328	(233.640, 461.407)	324.601	(229.072, 446.362)	-0.104	(−0.206, −0.001)	369.925	(261.904, 504.355)	360.016	(257.383, 487.581)	0.003	(−0.073, 0.079)
	81–84	347.488	(224.334, 497.944)	340.614	(225.241, 490.136)	0.126	(0.038, 0.214)	262.295	(164.448, 395.381)	257.351	(161.848, 387.697)	−0.005	(−0.123, 0.112)	299.636	(193.540, 441.986)	289.414	(186.036, 417.126)	0.102	(0.002, 0.201)
	85–89	247.006	(159.125, 353.751)	243.246	(160.335, 350.877)	0.12	(0.031, 0.209)	187.024	(117.726, 280.352)	183.719	(115.924, 277.569)	−0.003	(−0.122, 0.116)	208.943	(135.454, 309.077)	203.079	(130.745, 293.562)	0.079	(−0.020, 0.179)
	90–94	147.991	(95.258, 212.654)	145.49	(95.729, 210.724)	0.12	(0.030, 0.209)	112.283	(70.976, 168.284)	110.308	(69.883, 166.697)	0.002	(−0.118, 0.122)	122.134	(78.660, 182.646)	119.651	(76.819, 175.188)	0.052	(−0.042, 0.147)
	95+	48.805	(31.769, 70.813)	49.009	(32.231, 70.870)	0.111	(0.036, 0.186)	37.499	(23.553, 56.438)	37.333	(23.557, 56.439)	−0.012	(−0.116, 0.092)	39.639	(25.042, 59.513)	39.573	(25.068, 58.228)	0.013	(−0.066, 0.092)

### 3.3 DALY trends (1990–2021)

**Figure 7:**
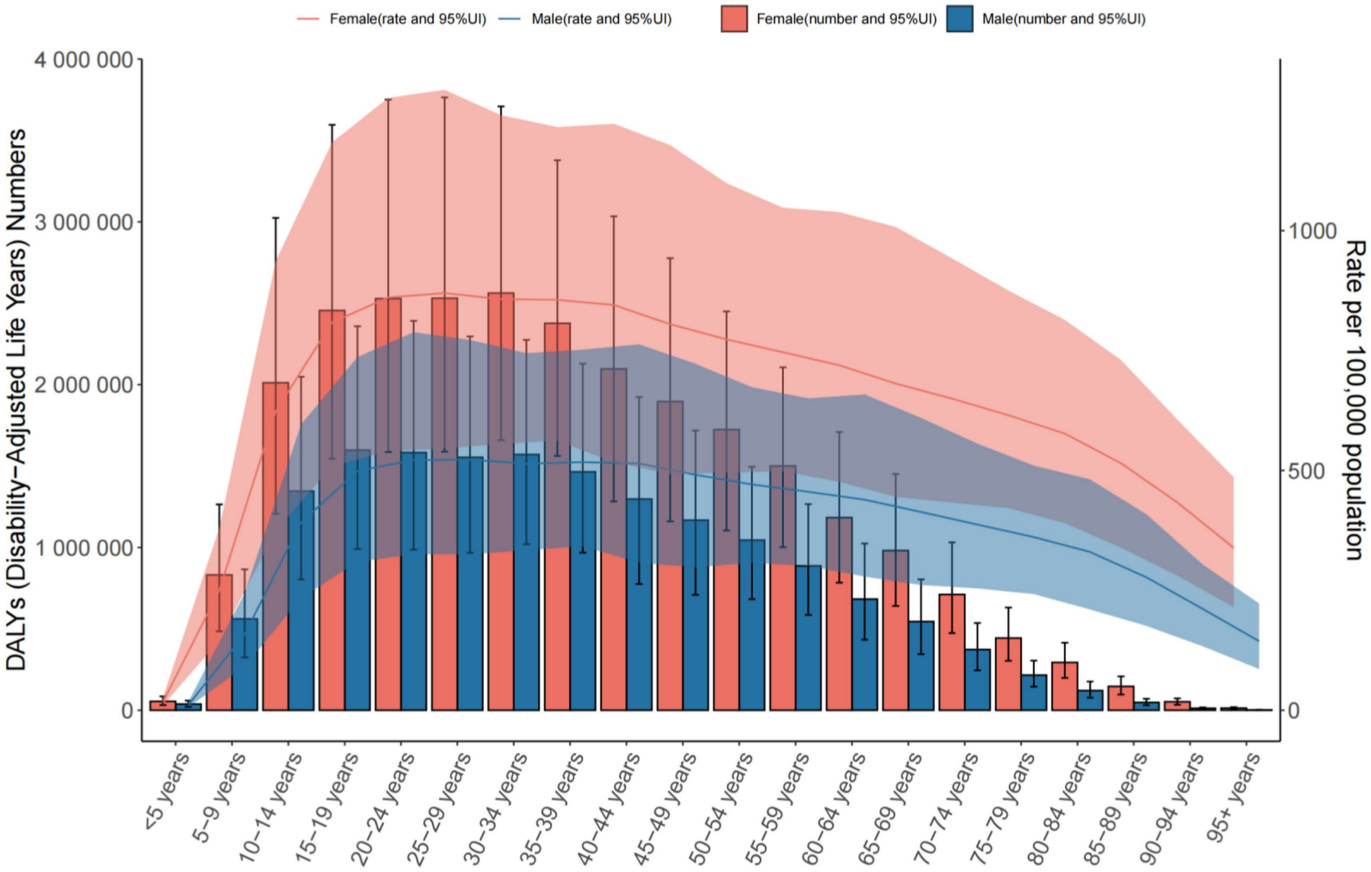
The number of DALYs and the rate of DALYs in anxiety disorders in the global from 1990 to 2021.

**Figure 8:**
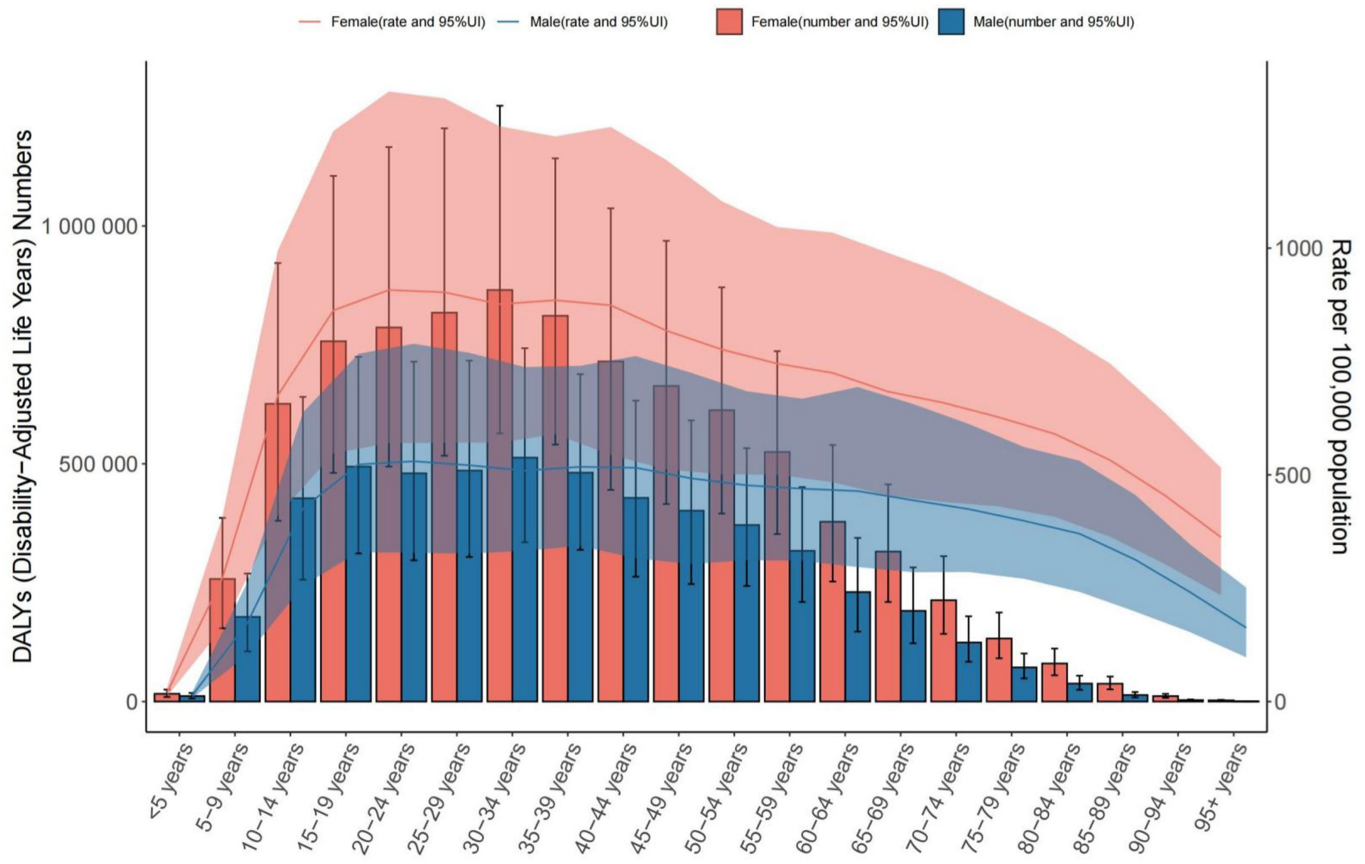
The number of DALYs and the rate of DALYs in anxiety disorders in middle SDI countries from 1990 to 2021.

**Figure 9:**
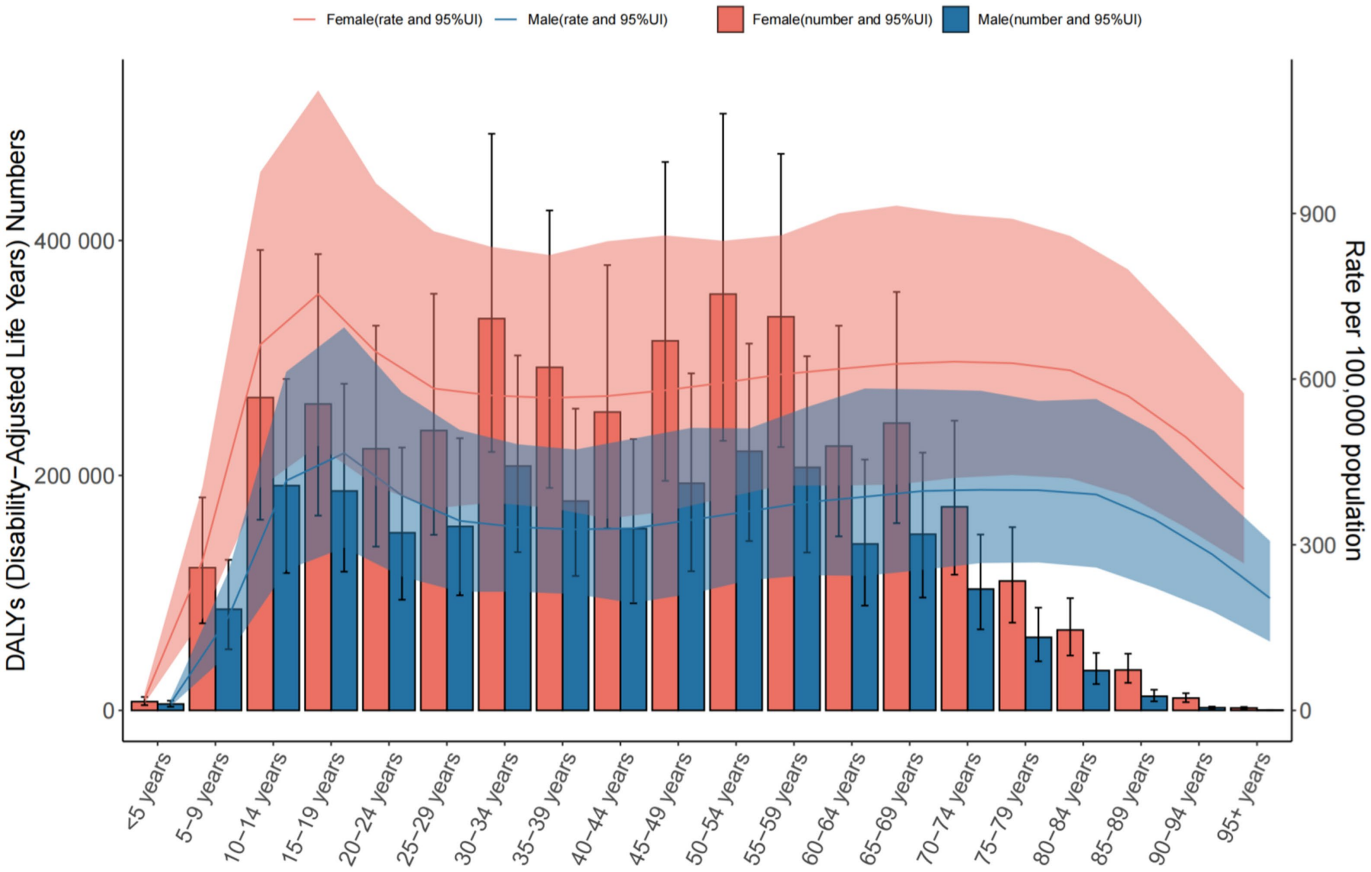
The number of DALYs and the rate of DALYs in anxiety disorders in China from 1990 to 2021.

Global: DALY rates increased from 431.165 to 538.686 per 100,000, highest in the 25–29 age group. Middle SDI Countries: DALY rates rose sharply, peaking in the 20–24 age group. China: DALY rates increased (416.160 to 443.821 per 100,000), highest in the 75–84 age group (Table 3 and Figures 7–9).

**Table 3:** Change trend of DALYs rates in different age groups for anxiety disorders in the global, SDI countries and China from 1990 to 2021.

District	Age group (years)	Male						Female						Both					
		1990		2021		AAPC		1990		2021		AAPC		1990		2021		AAPC	
		DALYs rate (1/100,000)	95%UI	DALYs rate (1/100,000)	95%UI	AAPC	95%CI	DALYs rate (1/100,000)	95%UI	DALYs rate (1/100,000)	95%UI	AAPC	95%CI	DALYs rate (1/100,000)	95%UI	DALYs rate (1/100,000)	95%UI	AAPC	95%CI
Global	<5	8.55	(4.966, 13.281)	11.185	(6.180, 17.481)	0.375	(0.176, 0.576)	12.992	(7.640, 20.005)	17.208	(9.985, 26.836)	0.524	(0.325, 0.724)	10.703	(6.277, 16.306)	14.097	(8.034, 22.149)	0.393	(0.190, 0.596)
	5–14	221.869	(134.422, 330.428)	273.216	(161.627, 414.615)	0.175	(0.003, 0.347)	349.899	(211.292, 515.485)	433.743	(259.996, 646.124)	0.201	(0.005, 0.397)	284.243	(171.705, 419.986)	350.949	(210.079, 525.680)	0.165	(−0.010, 0.340)
	15–19	419.679	(262.643, 625.109)	498.97	(309.220, 736.257)	0.122	(−0.040, 0.285)	674.531	(427.793, 990.262)	808.753	(508.675, 1184.243)	−0.13	(−0.303, 0.044)	545.057	(344.114, 811.645)	649.721	(407.894, 962.336)	0.112	(−0.050, 0.275)
	20–24	423.052	(264.935, 633.391)	521.75	(325.211, 788.324)	0.214	(0.029, 0.399)	697.877	(439.998, 1028.497)	860.747	(540.023, 1276.941)	−0.683	(−0.873, −0.493)	559.398	(353.323, 833.757)	688.508	(432.206, 1023.283)	0.19	(0.013, 0.367)
	25–29	422.062	(262.835, 618.569)	522.445	(325.091, 772.232)	0.272	(0.084, 0.460)	704.346	(442.656, 1047.770)	869.857	(545.851, 1293.687)	−0.951	(−1.198, −0.704)	562.432	(352.993, 836.327)	694.271	(430.966, 1027.149)	0.231	(0.053, 0.409)
	30–34	424.818	(270.901, 622.841)	514.12	(333.621, 744.562)	0.273	(0.096, 0.450)	709.71	(460.785, 1027.799)	857.247	(554.934, 1240.833)	−0.854	(−1.097, −0.609)	565.341	(363.901, 817.840)	683.805	(441.872, 984.562)	0.221	(0.064, 0.377)
	35–39	424.485	(278.077, 614.802)	517.395	(342.018, 751.987)	0.291	(0.124, 0.458)	701.406	(464.623, 986.634)	855.663	(562.323, 1216.096)	−0.632	(−0.828, −0.437)	560.847	(368.350, 795.596)	684.943	(449.650, 969.436)	0.241	(0.092, 0.389)
	40–44	431.494	(260.832, 645.636)	514.546	(307.476, 763.019)	0.236	(0.090, 0.382)	708.244	(434.020, 1032.415)	845.075	(517.037, 1222.983)	−0.42	(−0.579, −0.262)	566.956	(345.939, 833.964)	678.465	(413.105, 990.764)	0.192	(0.042, 0.343)
	45–49	428.268	(258.114, 638.805)	490.924	(298.111, 722.431)	0.155	(0.042, 0.267)	696.19	(426.644, 1019.437)	804.928	(492.408, 1178.421)	−0.376	(−0.544, −0.207)	559.578	(348.751, 823.663)	647.19	(402.783, 944.419)	0.121	(−0.008, 0.251)
	50–69	403.883	(266.552, 577.810)	448.836	(296.966, 638.794)	0.094	(−0.002, 0.191)	657.563	(449.124, 929.206)	735.723	(498.776, 1031.771)	−0.599	(−0.836, −0.362)	532.425	(361.120, 747.084)	595.114	(404.255, 835.013)	0.045	(−0.074, 0.164)
	70–74	362.589	(239.705, 526.506)	387.157	(254.109, 555.962)	0.026	(−0.055, 0.108)	607.553	(402.484, 879.861)	649.786	(432.913, 942.324)	−0.695	(−0.965, −0.424)	498.705	(331.053, 719.817)	526.801	(351.567, 756.204)	−0.14	(−0.239, −0.042)
	75–79	337.665	(227.008, 472.208)	361.107	(242.146, 510.204)	0.062	(0.003, 0.121)	589.787	(406.416, 842.006)	615.21	(421.465, 875.196)	−0.602	(−0.854, −0.349)	486.437	(335.708, 696.469)	500.018	(343.081, 704.940)	−0.154	(−0.241, −0.067)
	80–84	306.331	(197.799, 451.744)	330.06	(210.118, 481.395)	0.129	(0.092, 0.166)	555.939	(382.095, 788.793)	576.849	(390.202, 813.950)	−0.478	(−0.712, −0.244)	462.213	(318.954, 650.216)	473.572	(321.371, 667.018)	−0.134	(−0.205, −0.063)
	85–89	259.394	(164.135, 387.760)	277.27	(175.926, 408.808)	0.163	(0.120, 0.206)	498.247	(329.785, 707.177)	515.111	(339.090, 730.401)	−0.37	(−0.601, −0.139)	418.202	(272.735, 593.529)	425.364	(274.402, 602.171)	−0.108	(−0.176, −0.040)
	90–94	201.499	(126.982, 295.990)	211.322	(133.423, 303.920)	0.169	(0.124, 0.214)	423.277	(272.035, 600.907)	433.287	(280.647, 606.129)	−0.269	(−0.496, −0.042)	358.12	(229.255, 502.466)	360.969	(232.445, 506.553)	−0.081	(−0.132, −0.031)
	95+	141.841	(84.147, 217.933)	143.946	(85.900, 222.636)	0.132	(0.085, 0.179)	341.33	(215.207, 492.434)	338.598	(215.307, 485.725)	−0.232	(−0.409, −0.055)	290.339	(182.885, 423.336)	284.597	(180.529, 409.607)	−0.148	(−0.190, −0.107)
Middle SDI	<5	9.789	(5.883, 14.917)	12.68	(7.182, 19.628)	0.247	(0.039, 0.454)	14.352	(8.577, 21.998)	19.386	(11.487, 29.716)	0.411	(0.217, 0.606)	11.988	(7.277, 18.371)	15.892	(9.281, 24.309)	0.34	(0.140, 0.540)
	5–14	246.706	(151.904, 361.804)	298.402	(179.266, 437.338)	0.061	(−0.140, 0.262)	375.207	(228.293, 548.273)	472.047	(286.073, 689.447)	0.183	(−0.177, 0.544)	309.295	(188.258, 451.510)	381.723	(230.081, 557.194)	0.133	(−0.064, 0.331)
	15–19	421.565	(266.409, 623.552)	529.984	(328.052, 789.107)	0.028	(−0.145, 0.200)	681.073	(432.735, 1002.398)	907.644	(570.134, 1345.371)	0.207	(0.033, 0.381)	550.124	(347.788, 814.986)	714.652	(446.231, 1058.821)	0.121	(−0.050, 0.292)
	20–24	441.795	(279.658, 650.502)	522.848	(329.713, 767.029)	0.182	(−0.017, 0.381)	686.046	(440.235, 1002.072)	862.667	(547.402, 1258.303)	0.331	(0.132, 0.530)	562.01	(358.450, 831.514)	686.556	(437.692, 1013.995)	0.27	(0.079, 0.461)
	25–29	408.29	(256.970, 599.712)	521.184	(326.133, 769.190)	0.318	(0.114, 0.522)	673.003	(423.846, 1003.021)	902.624	(570.705, 1330.447)	0.411	(0.207, 0.615)	539.634	(338.984, 802.274)	709.199	(446.990, 1041.237)	0.383	(0.193, 0.574)
	30–34	408.885	(262.588, 592.135)	509.272	(332.633, 737.590)	0.377	(0.168, 0.587)	677.086	(438.185, 976.211)	876.343	(571.100, 1268.726)	0.441	(0.283, 0.599)	540.268	(348.825, 781.963)	690.985	(455.292, 997.073)	0.426	(0.265, 0.587)
	35–39	408.394	(263.708, 582.651)	517.732	(343.144, 740.503)	0.415	(0.206, 0.626)	663.998	(433.903, 936.238)	885.11	(589.672, 1246.367)	0.487	(0.336, 0.638)	533.275	(346.960, 754.153)	700.097	(463.787, 982.444)	0.473	(0.319, 0.627)
	40–44	418.524	(256.495, 621.683)	515.646	(316.041, 761.826)	0.337	(0.170, 0.504)	674.902	(414.890, 980.585)	873.909	(543.799, 1267.169)	0.385	(0.196, 0.574)	542.221	(333.131, 792.242)	693.456	(433.081, 1008.280)	0.388	(0.232, 0.544)
	45–49	421.616	(257.042, 620.897)	492.289	(303.098, 725.049)	0.22	(0.100, 0.341)	668.379	(419.052, 975.898)	818.133	(512.130, 1194.416)	0.251	(0.065, 0.437)	540.438	(338.201, 782.495)	654.777	(413.301, 949.315)	0.268	(0.132, 0.404)
	50–69	422.589	(281.592, 598.912)	466.22	(309.734, 660.014)	0.029	(−0.078, 0.137)	644.907	(436.830, 901.493)	739.414	(497.352, 1025.789)	0.035	(−0.144, 0.215)	531.993	(358.716, 739.782)	605.538	(408.162, 839.371)	0.059	(−0.072, 0.191)
	70–74	400.213	(269.356, 570.256)	424.481	(285.776, 612.414)	−0.089	(−0.184, 0.006)	606.276	(403.104, 877.349)	658.892	(440.224, 944.850)	−0.192	(−0.366, −0.018)	510.765	(342.132, 728.982)	547.514	(369.590, 779.238)	−0.167	(−0.300, −0.033)
	75–79	376.34	(257.250, 526.844)	398.617	(270.788, 561.335)	−0.027	(−0.087, 0.033)	584.501	(402.504, 838.895)	626.779	(430.138, 884.909)	−0.196	(−0.354, −0.038)	493.193	(341.210, 695.490)	521.871	(358.611, 732.177)	−0.168	(−0.285, −0.051)
	80–84	337.967	(222.835, 493.538)	370.198	(241.846, 531.144)	0.125	(0.077, 0.174)	550.252	(382.871, 774.133)	590.389	(407.142, 821.639)	−0.125	(−0.269, 0.020)	462.74	(315.712, 645.287)	495.621	(342.701, 697.842)	−0.078	(−0.191, 0.036)
	85–89	279.121	(180.803, 412.775)	313.15	(198.560, 456.141)	0.294	(0.242, 0.347)	493.38	(327.474, 708.307)	532.399	(363.542, 745.510)	−0.015	(−0.143, 0.113)	409.706	(269.680, 579.191)	448.03	(303.154, 625.440)	0.071	(−0.042, 0.184)
	90–94	210.029	(133.255, 304.031)	240.712	(152.737, 345.630)	0.464	(0.422, 0.506)	412.559	(265.540, 587.262)	453.675	(302.405, 635.637)	0.136	(0.040, 0.233)	336.608	(215.368, 474.265)	381.333	(253.297, 530.155)	0.292	(0.208, 0.375)
	95+	143.895	(86.297, 222.776)	162.763	(97.496, 251.545)	0.438	(0.421, 0.455)	319.868	(200.667, 470.378)	362.315	(234.255, 515.375)	0.331	(0.278, 0.384)	260.656	(163.268, 385.232)	298.341	(192.639, 430.225)	0.4	(0.364, 0.435)
China	<5	11.046	(6.547, 16.761)	13.062	(7.378, 19.818)	0.25	(−0.012, 0.513)	17.367	(10.467, 26.227)	20.8	(12.382, 31.849)	0.524	(0.325, 0.724)	14.017	(8.490, 21.188)	16.653	(9.863, 25.224)	0.407	(0.189, 0.626)
	5–14	278.973	(173.344, 407.447)	286.273	(173.651, 422.992)	0.043	(−0.331, 0.419)	441.125	(269.763, 637.503)	456.115	(277.907, 672.052)	0.201	(0.005, 0.397)	357.103	(219.391, 517.673)	365.637	(222.947, 537.761)	0.094	(−0.267, 0.456)
	15–19	454.898	(292.052, 667.075)	465.971	(294.677, 693.781)	−0.202	(−0.349, −0.055)	715.104	(463.532, 1052.588)	754.067	(479.381, 1123.012)	−0.13	(−0.303, 0.044)	581.511	(376.707, 854.701)	599.422	(382.082, 891.366)	−0.202	(−0.370, −0.033)
	20–24	387.936	(248.057, 568.554)	389.073	(242.464, 575.749)	−0.564	(−0.737, −0.390)	629.817	(404.649, 923.013)	649.182	(406.452, 954.594)	−0.683	(−0.873, −0.493)	506.103	(325.394, 737.229)	511.021	(319.850, 754.802)	−0.652	(−0.817, −0.487)
	25–29	343.691	(215.176, 500.051)	343.316	(214.785, 507.910)	−0.669	(−0.973, −0.363)	573.587	(363.955, 843.228)	583.015	(365.872, 867.871)	−0.951	(−1.198, −0.704)	455.539	(285.497, 667.531)	456.584	(282.606, 680.571)	−0.833	(−1.069, −0.597)
	30–34	326.006	(207.198, 472.453)	331.919	(214.923, 482.027)	−0.513	(−0.827, −0.198)	550.543	(361.414, 797.189)	570.447	(376.378, 839.546)	−0.854	(−1.097, −0.609)	433.434	(279.638, 626.296)	447.034	(294.243, 654.182)	−0.707	(−0.944, −0.470)
	35–39	326.294	(206.994, 466.732)	327.797	(210.514, 472.462)	−0.322	(−0.586, −0.057)	547.779	(359.103, 783.196)	566.029	(367.074, 824.867)	−0.632	(−0.828, −0.437)	433.262	(280.955, 617.030)	443.8	(286.997, 642.776)	−0.499	(−0.675, −0.324)
	40–44	331.835	(200.127, 494.485)	329.814	(194.430, 492.244)	−0.141	(−0.371, 0.089)	555.056	(342.116, 816.899)	569.381	(347.255, 849.724)	−0.42	(−0.579, −0.262)	438.013	(268.021, 645.011)	446.565	(269.685, 667.168)	−0.293	(−0.413, −0.173)
	45–49	344.247	(205.339, 505.438)	344.891	(211.358, 511.802)	−0.144	(−0.362, 0.075)	562.777	(354.714, 825.306)	579.814	(360.179, 860.377)	−0.376	(−0.544, −0.207)	447.461	(279.455, 648.662)	460.434	(288.446, 683.019)	−0.256	(−0.387, −0.126)
	50–69	375.853	(248.811, 538.074)	377.723	(247.985, 540.264)	−0.389	(−0.604, −0.173)	596.612	(404.969, 836.982)	609.65	(409.710, 862.455)	−0.599	(−0.836, −0.362)	482.288	(325.687, 677.193)	493.604	(330.802, 697.487)	−0.486	(−0.673, −0.298)
	70–74	394.926	(267.288, 563.769)	399.481	(266.836, 579.027)	−0.346	(−0.493, −0.199)	618.159	(409.645, 886.264)	631.609	(421.101, 898.823)	−0.695	(−0.965, −0.424)	514.651	(345.013, 733.483)	518.993	(350.189, 733.760)	−0.598	(−0.801, −0.395)
	75–79	392.009	(267.674, 551.320)	398.837	(267.970, 560.564)	−0.212	(−0.293, −0.132)	614.649	(424.733, 872.372)	628.878	(426.542, 890.475)	−0.602	(−0.854, −0.349)	518.849	(361.713, 726.725)	520.505	(353.017, 725.589)	−0.532	(−0.715, −0.350)
	80–84	381.189	(251.313, 556.978)	391.128	(258.731, 564.015)	−0.076	(−0.143, −0.008)	603.02	(416.776, 849.112)	615.926	(420.388, 859.372)	−0.478	(−0.712, −0.244)	517.596	(350.746, 728.183)	517.395	(356.706, 725.204)	−0.424	(−0.602, −0.246)
	85–89	341.005	(218.980, 502.732)	346.473	(222.317, 506.155)	0.022	(−0.089, 0.134)	560.722	(369.130, 796.727)	569.518	(388.399, 798.767)	−0.37	(−0.601, −0.139)	489.264	(321.360, 694.390)	488.012	(327.260, 684.887)	−0.325	(−0.520, −0.130)
	90–94	275.844	(174.763, 395.380)	283.005	(179.972, 404.172)	0.13	(−0.032, 0.292)	485.828	(313.943, 685.892)	494.839	(331.091, 689.250)	−0.269	(−0.496, −0.042)	430.065	(280.250, 606.373)	436.402	(287.381, 611.335)	−0.22	(−0.449, 0.010)
	95+	207.541	(125.286, 316.443)	203.201	(124.685, 306.791)	0.22	(0.027, 0.414)	408.993	(265.440, 593.587)	401.137	(266.196, 574.217)	−0.232	(−0.409, −0.055)	370.34	(240.785, 535.919)	363.679	(241.102, 523.064)	−0.178	(−0.375, 0.020)

### 3.4 Projections for China (2022–2035)

Projections for 2022–2035 indicate: Prevalence: An overall increase from 3,467.02 to 3,551.57 per 100,000, driven by rising rates among females (4,396.28 to 4,575.95 per 100,000) despite a slight decline in males (2,569.21 to 2,552.09 per 100,000). Incidence: An overall increase from 539.93 to 544.88 per 100,000, with female incidence rising (647.97 to 660.54 per 100,000) and male incidence declining (444.63 to 442.63 per 100,000). DALYs: An overall increase from 418.02 to 429.61 per 100,000, reflecting rising female DALYs (528.76 to 552.55 per 100,000) and a marginal decline in males (313.14 to 311.94 per 100,000).

## 4 Discussion

Anxiety disorders are mental illnesses characterized by anxiety as the core feature, falling within the category of neuroses, with clinical manifestations including generalized, persistent anxiety or recurrent episodes of panic and unease (6). Various factors may trigger anxiety, including social-environmental factors such as exam pressure among students (7), family relationships, and socioeconomic conditions (8). Specific medical conditions, such as cancer (9), epilepsy (10), and major infectious diseases (11), not only provoke significant anxiety but also substantially reduce patients’ quality of life, creating a vicious cycle. Additionally, physiological changes during pregnancy (12) and menopausal syndrome contribute to anxiety. Bibliometric studies show a steady annual increase in anxiety-related publications, with research hotspots focusing on cognitive dysfunction, risk factors, comorbidity mechanisms, neuroimaging, and intervention strategies (13).

This study reveals that from 1990 to 2021, the global number of anxiety disorder cases and prevalence rates have risen. In 2021, the 30–34 age group had the highest number of anxiety cases worldwide (male-to-female ratio ≈ 3:7), and the global incidence and burden of anxiety increased over this period. Similarly, the number and rate of disability-adjusted life years (DALYs) due to anxiety rose globally, with the 30–34 age group showing the highest DALYs in 2021, while the 25–29 age group peaked in DALYs rate (694.271 per 100,000, 95% UI = 430.966–1027.149).

In middle Socio-Demographic Index (SDI) countries, anxiety cases and prevalence also increased from 1990 to 2021, with the 30–34 age group being the most affected (male-to-female ratio ≈ 4:7), alongside rising incidence and DALYs trends. By 2021, the 30–34 age group had the highest DALYs (male-to-female ratio ≈ 3:5), while the 20–24 age group reached a DALYs rate of 1,266,663.634 per 100,000 (95% UI = 790,909.401–1,876,675.377).

In contrast, China exhibited a declining trend in anxiety cases and prevalence from 1990 to 2021, with the 50–54 age group having the highest case numbers (male-to-female ratio ≈ 3:5). Despite a decline in incidence, DALYs numbers and rates still increased. In 2021, the 50–54 age group had the highest DALYs (male-to-female ratio ≈ 3:6), while the 75–84 age group peaked at a DALYs rate of 599.422 per 100,000 (95% UI = 382.082–891.366).

Globally and in middle SDI countries, the 30–34 age group bore the heaviest anxiety burden in 2021, with female cases approximately double those of males, closely linked to pregnancy in this age range. A cross-sectional study indicated that early pregnancy stress is a significant risk factor for maternal anxiety (OR = 6.428, 95% CI: 4.753–8.694) (14), with notable correlations between prenatal and postnatal psychiatric symptoms (15). Factors influencing maternal anxiety include workplace discrimination (e.g., limited promotions, reduced income during maternity leave) and concerns over childcare costs (e.g., education expenses) (16). Furthermore, hormonal fluctuations are strongly tied to anxiety. Post-pregnancy, progesterone and estrogen levels surge, directly affecting neurotransmitter secretion (e.g., dopamine, serotonin). Progesterone may induce anxiety and emotional instability by inhibiting GABA receptor activity (17), while drastic estrogen fluctuations heighten amygdala sensitivity, making pregnant women more prone to tension and fear (18). Human chorionic gonadotropin spikes in early pregnancy, and its metabolites may act on the central nervous system, causing nausea and vomiting that indirectly exacerbate anxiety (19). Hormonal changes also trigger physical symptoms like morning sickness, frequent urination, and acid reflux (20), amplifying negative emotions via a “somatic-emotional feedback loop.” For instance, persistent vomiting may lead to nutritional imbalances, further disrupting neurotransmitter synthesis and forming a vicious cycle of “physical discomfort → low mood → worsening symptoms” (21). Maternal physical and mental health requires multidimensional care, such as mindfulness meditation to reduce amygdala activity, regular exercise to boost endorphin secretion, enhanced spousal involvement in childcare at the family level, and improved paid maternity leave policies. In summary, pregnancy-related anxiety is a complex interplay of biological, psychological, and social factors. Hormonal fluctuations directly affect emotional centers via neuroendocrine pathways, while social pressures exacerbate psychological burdens through structural inequalities. Future research should explore gene–environment interactions and establish a “perinatal mental health ecological support system” through cross-sector collaboration, shifting from “disease treatment” to “full-cycle health promotion.”

DALYs, which combine years of life lost (YLLs) and years lived with disability (YLDs) to quantify disease burden, peaked in the 25–29 age group globally and the 20–24 age group in middle SDI countries. In middle SDI countries, socioeconomic factors such as low income, low education, and declining fertility rates worsened the rise in DALYs rates among youth. Millennials face mounting pressures from rising youth unemployment, reduced social mobility, and housing cost crises, delaying their transition to independent adulthood and intensifying anxiety burdens in the 25–29 age group (22). Specifically, prolonged unemployment elevates stress hormone levels (e.g., cortisol), triggering anxiety and depression, thus increasing DALYs linked to mental disorders. Unemployment also worsens the course and prognosis of pre-existing mental conditions (23). Additionally, a disconnect between education systems and labor market demands traps youth in a “high education-low income” dilemma (24), driving economic stress and high-risk behaviors (e.g., alcohol or drug abuse), indirectly elevating DALYs rates.

Epidemiological Trends of Anxiety Disorders (2022–2035) reveal significant gender disparities and an overall rise in disease burden, a phenomenon that warrants multidimensional analysis from social, biological, and public health perspectives. The data indicate a persistent increase in the prevalence, incidence, and disability-adjusted life years (DALYs) of anxiety disorders among females, while males show slight declines or stagnation. For instance, the prevalence among females is projected to rise from 4,396.28 to 4,575.95 per 100,000, whereas male prevalence decreases from 2,569.21 to 2,552.09 per 100,000. This divergence may stem from multiple factors: Sociocultural Pressures: Women often face heightened role conflicts (e.g., dual burdens of career and family responsibilities), risks of gender-based violence, and sensitivity to societal evaluations of appearance, all of which may exacerbate anxiety. Biological Mechanisms: Hormonal fluctuations (e.g., menstrual cycles, pregnancy, and menopause) may influence women’s susceptibility to anxiety. Research suggests interactions between estrogen and the GABA system could modulate anxiety responses. Gender Differences in Healthcare-Seeking Behavior: Men may underreport symptoms or avoid treatment due to societal stereotypes of “masculinity,” leading to underestimation in statistical data. In contrast, women’s higher healthcare utilization rates may partially explain their rising incidence. Despite minor improvements in male metrics, the overall prevalence, incidence, and DALYs of anxiety disorders continue to climb, signaling heightened challenges for public health systems. The increase in DALYs from 418.02 to 429.61 per 100,000 reflects the profound impact of anxiety disorders on individual functioning and quality of life. The marked rise in female DALYs (528.76 → 552.55/100,000) compared to males may be linked to their higher comorbidity rates (e.g., depression, somatization disorders) and disruptions in social roles (e.g., occupational withdrawal). This trend underscores that anxiety disorders are not merely psychological issues but also a major source of socioeconomic loss—manifesting as reduced productivity, increased healthcare costs, and heavier family caregiving burdens.

## 5 Conclusion

This study aimed to assess the disease burden of anxiety disorders globally, in middle SDI countries, and in China from 1990 to 2021 using the GBD database. Globally and in middle SDI countries, prevalence, incidence, and DALY rates increased, with the 30–34 age group showing the highest case burden (women outnumbering men) and peak DALY rates in younger adults (25–29 globally, 20–24 in middle SDI). In contrast, China saw a decline in prevalence and incidence, with the burden peaking later (50–54 for cases, 75–84 for DALYs). Projections suggest a rising trend in China by 2035, highlighting the need for targeted interventions across regions and age groups.

## Data Availability

The original contributions presented in the study are included in the article/supplementary material, further inquiries can be directed to the corresponding author.

## Footnotes

^1^ https://vizhub.healthdata.org/gbd-results/

^2^ https://medhub.shinyapps.io/ShinyGBD/

^3^ https://folk.ntnu.no/andrerie/software.html
